# Expansion of the role of midwives in gender equity and sexual/reproductive health issues in Japan

**DOI:** 10.4069/kjwhn.2021.08.24.1

**Published:** 2021-09-30

**Authors:** Yoko Shimpuku

**Affiliations:** Graduate School of Biomedical and Health Sciences, Hiroshima University, Hiroshima, Japan

## Issues regarding the low fertility rate in Japan

In terms of an aging society, Japan ranks the first among the 38 member countries of the Organization for Economic Co-operation and Development (OECD), with the highest proportions of the population in the ≥65 years (27.7%) and ≥80 years (8.5%) age groups in 2019 [1]. According to the Ministry of Health, Labour and Welfare of Japan, the fertility rate in Japan decreased gradually from 2.16 children per woman in 1971 to 1.36 children per woman in 2019 [2]. The number of recorded births in 2020 was 840,832, which was 24,407 less than that in 2019 [3]. There is also an increasing trend of women preferring to conceive at an older age. The average age of women at their first delivery increased from 29.1 years in 2005 to 30.7 years in 2019, thereby influencing their decisions regarding subsequent pregnancies [4]. Further, the coronavirus disease 2019 (COVID-19) pandemic has been considered a factor contributing to the decreasing birth rate [5].

The number of births using assisted reproductive technology (ART) increased after the introduction of advanced ART techniques, from 21,704 in 2008 to 56,979 in 2018 [6]. This treatment is covered by insurance only when the causes of infertility are identified and treated. Advanced treatments, such as artificial insemination by husband, in vitro fertilization (IVF), and intracytoplasmic sperm injection, are not covered and are mostly paid out of pocket due to limited funding from local governments. Women and their families have been struggling, not only with the complications and side effects of treatment, but also with its high costs, as the cost of frozen embryo transfer ranges from ¥210,000 to ¥980,000 (roughly 2,000-9,000 US dollars). Until the end of 2020, there was a subsidy of ¥150,000 (except for the first time of treatment [¥300,000]), while the current subsidy is ¥300,000 (roughly 2,800 US dollars) [7].

A recent study revealed that among the 513 Japanese women who received advanced infertility treatment, 54% showed symptoms of depression, with the rate having increased by nearly 80% when they were in their 20s [8]. The researchers pointed out that high costs may have contributed to the symptoms [8]. In 2020, the Suga administration announced that infertility treatment will be covered by health insurance starting in April 2022, either through employees’ health insurance or through the national health insurance program for self-employed individuals and people out of employment [9]. In June 2021, the Japan Society for Reproductive Medicine published the Reproductive Medicine Guideline [10] to recommend which treatments should be covered by insurance. This guideline strongly recommends the inclusion of IVF and medical treatment for males, including treatment for erectile dysfunction. In addition, it also recommends a preimplantation examination for women who have experienced miscarriage twice, and prescription of antidepressants for patients with ejaculation disorder. Based on this guideline, experts are expected to discuss healthcare fees at the Central Social Insurance Medical Council in early 2022. Until then, the subsidy for treatment has increased, and the upper limit of household income to receive the subsidy was abolished. Apart from health insurance, the social support system, such as coworkers and other people surrounding women affected by infertility, also plays a significant role, as treatment often requires multiple visits to clinics/hospitals to receive testing of hormone levels and to receive medications, including injections.

## Insufficient support during the postpartum period

Another important issue in Japan that requires more attention is postpartum depression. A recent retrospective cohort study using the Diagnosis Procedure Combination database, a national database on acute-care inpatients in Japan, reported that the prevalence of suicide attempts was significantly higher among postpartum women (6.2%) than among pregnant women (0.7%; *p*<.001) and that postpartum patients were more likely to have depression [11]. In fact, suicide is the leading cause of maternal death in Japan [12]. During the COVID-19 pandemic, social support for pregnant women and mothers has decreased. Mothering or parenting classes were canceled, and home visits a month after birth by a public health nurse or midwife are conducted only within a very short period. Furthermore, parents with small children have avoided leaving their homes due to fear of infection. Given these circumstances that limit social interactions with their support systems, mothers do not receive the adequate support they need and deserve.

In Japan, mandatory maternity leave, called postpartum leave (*sango kyuka*), lasts for 8 weeks [13]. Mothers also have the option to avail themselves of childcare leave (*ikuji kyuka*) after postpartum leave, depending on the policies of the employers [13]. Postpartum leave is a paid leave, and they can receive childcare leave with benefits at 67% of their salary, with the exemption of some payments for social security funds [13]. Therefore, the decrease in income during leaves remains approximately 20% [13]. On June 3, 2021, the Amendment of the Childcare and Nursing Care Leave Law was passed and approved by the Diet (the parliament of Japan) [14]. This will allow men to avail themselves of leave for 4 to 8 weeks after the birth of the child [14]. There have been serious concerns regarding how to improve mothers’ experiences of “One Operation” (meaning one person, usually a mother, assumes all household and childcare responsibilities), as currently only 12.65% of Japanese fathers take paternity leave [15]. Men utilize approximately 83 minutes of average daily hours a week for household chores, childcare, and elderly care [16]. Among OECD countries, the disparity between women and men with respect to unpaid working hours is highest in Japan, with women spending 5.5 times more hours on unpaid work than men [17]. Therefore, in addition to implementing this important policy for men to take paternity leave, it is necessary to adequately provide learning opportunities for new fathers with respect to childcare so that they can understand how to appropriately begin their parenting journey with a new mother. As midwives, we often say, “*Provide midwifery care for every woman.*” However, in many cases, and bearing in mind the difficulties that exist, we should be more inclusive in considering the father’s role as well, and instead recognize that midwives are well-poised to “*Provide care for every woman and man: every family.*”

## Continuing gender disparities in Japan

In Japan, women with children face difficulties in advancing their careers. Despite the government’s promise that they would hire more women in decision-making positions, such as in the parliament, management or executive positions in organizations, and professions with a high level of specialization, to be at 30% by the year 2020, with the slogan of “202030,” the role of working mothers in these positions remains low [18]. The Diet (Japanese parliament) consists of only 9.9% women, with 10.8% in management positions and 6.2% in executive positions [19]. The percentage of female professors was 17.7% in 2020 [19]. It has been called the “mummy’s track” when women are considered separately from the regular career ladder and are not assigned to more important roles or are not promoted after having a baby. Once a woman quits a job, it is often difficult to return to full-time employment. Among women who desire to work and are currently unemployed, their top reason for not having entered the workforce is due to childbirth and childcare [20]. There is a substantial income disparity, as illustrated by the fact that women in Japan were paid only 73.3% of what their male counterparts earned in 2018 [21]. Nurseries or daycare centers are another factor that has not been addressed for many years, as many women are not sure if they can find a nursery for their child when they return to work. Thus, these gender disparities in society tend to discourage women from conceiving at a younger age.

## Pregnancy and childbirth experience

Midwives in Japan have provided quality care and support during pregnancy and childbirth. Coincidentally, I was pregnant when I was asked to author this article. I was able to go through pregnancy, childbirth, and parenting amidst the COVID-19 pandemic. Drake [22] stated the importance of storytelling, and thus I would like to share my story of how I positively experienced midwifery care in Japan. During antenatal care, most healthcare facilities provide mothers with the opportunity to have discussions with a midwife, aside from the physical consultation with the obstetrician. As most mothering or parenting classes were canceled because of the COVID-19 pandemic, it was a valuable time for pregnant women to ask questions and talk about their everyday lives. In my experience, I had itchy rashes due to pregnancy (prurigo gestationis), but I did not mention it during my obstetric consultation as I knew that it was because of my pregnancy and that it would eventually disappear after delivery. However, a midwife noticed these rashes and asked me if I took or applied any medication to treat the rashes. When I said no, she asked the obstetrician for a prescription, which relieved my stress from itchiness. Furthermore, it is important to be aware of the hospital environment before delivery, similar to when pregnant women usually visit the labor and delivery rooms during their mothering or parenting classes. Because visiting these rooms during pregnancy was not allowed due to the COVID-19 pandemic, I asked a midwife to provide images of the labor and delivery rooms and asked how time during labor is spent there. It was especially important for me to have a clear perspective on several aspects prior to actual labor and delivery: for example, room décor (if I can relax or not), decision-making in case of complications, equipment I may use during labor, and location of the operation theater in case of an emergency. Although I am a midwife who knows the general principles of pregnancy, labor, and delivery, having another midwife present with me during the process helped me better understand my concerns and prepare for the journey postpartum. I cannot describe all the details regarding the delivery of my child because of the page limit here, but overall, I faced anxiety and experienced tough labor pain, similar to those experienced by other pregnant women. Thankfully, a midwife was always with me to encourage me, provide respectful care, and deliver my baby safely. I will never forget the moment she brought my baby to my side.

## Expansion of the roles of midwives

It is essential to discuss the vitally important role of midwives in addressing wider social issues, such as gender, sexual, and reproductive health, through multidisciplinary research and policymaking. To reiterate, I believe that midwives play an important role in pregnancy and childbirth with respect to the quality of care they provide. In general, women in Japan face multiple difficulties when they decide to have a child. Having said this, midwives need to expand their roles in research and policy advocacy in these areas, including promotion of gender equity and policymaking for women; otherwise, we cannot expect the low fertility rate to improve.

As mentioned earlier, women are vulnerable to depression during infertility treatment and the postpartum period. For infertility treatment, certified courses in infertility nursing started in 2003 in Japan for post-graduate specialty nurses, but the number of certified nurses in this specialty is limited, especially in local areas. As midwives in hospital settings often encounter women receiving infertility treatment, they need to strengthen their capacities in practice and research in this area so that women’s needs are reflected in practice and brought to the policy level.

Regarding postpartum care, fathers and other members of the family also faced difficulties in learning proper caretaking skills because many family members were not allowed to visit the hospital, let alone attend births, during the COVID-19 pandemic in Japan. To increase support for women in the postpartum period, midwifery postpartum care (*sango* care) has become more important. In Asian countries, it is common to stay in care centers after birth. In Korea, these centers are called *sanhujoriwon*. In Japan, in *sango* care, midwives are the main care providers for postpartum women and are reimbursed by the government. This was highlighted in the Revised Maternal and Child Health Law in 2019, and *sango* care became more affordable as it was funded by the local governments [23]. It is an excellent example of the Japanese Midwives Association working with other stakeholders to effectively provide families with midwifery care, alleviating serious financial concerns. The next step is to explore how midwives can teach and involve fathers in postpartum care.

The Committee of Empowering Early Career Scientists of the Japan Academy of Midwifery, of which I am the co-leader, hosted a seminar regarding changing postpartum care to promote fathers’ involvement in September 2021. We invited a midwife, a psychologist, and a chief executive officer of a home help company to discuss the issue at hand from different points of view. In addition to this event, we have been conducting seminars on emerging topics in collaboration with young researchers in different disciplines and other stakeholders so that the audience, typically midwives in clinical areas, will learn the importance of a multidisciplinary approach.

To solve these issues at the social level, it is important to strengthen the research capabilities of midwives and discuss issues with different professions so that innovative ideas emerge and we can find solutions to address the current issues in society. The year 2020 was the Year of the Nurse and Midwife, which encouraged our leadership in society. Midwives can show their leadership in the above-mentioned new areas and offer professional services that make a lasting impact on women, men, and families.
